# Violência obstétrica à luz da Teoria da Diversidade e Universalidade do Cuidado Cultural**

**DOI:** 10.15649/cuidarte.1536

**Published:** 2022-08-08

**Authors:** Bruna Larisse Pereira Lima Melo, Felice Teles Lira dos Santos Moreira, Rayane Moreira de Alencar, Beatriz de Castro Magalhães, Edilma Gomes Rocha Cavalcante, Evanira Rodrigues Maia, Grayce Alencar Albuquerque

**Affiliations:** 1 Universidade Regional do Cariri (URCA), Farias Brito-CE, Brasil. E-mail: bruna_la_risse@hotmail.com Universidade Regional do Cariri Universidade Regional do Cariri Farias Brito CE Brazil bruna_la_risse@hotmail.com; 2 Universidade Regional do Cariri (URCA), Crato-CE, Brasil. E-mail: felicelira@hotmail.com Universidade Regional do Cariri Universidade Regional do Cariri Crato CE Brazil felicelira@hotmail.com; 3 Universidade Regional do Cariri (URCA), Crato-CE, Brasil. E-mail: rayanealencar@hotmail.com Universidade Regional do Cariri Universidade Regional do Cariri Crato CE Brazil rayanealencar@hotmail.com; 4 Universidade Regional do Cariri (URCA), Crato-CE, Brasil. E-mail: beatriz.castro022015@gmail.com Universidade Regional do Cariri Universidade Regional do Cariri Crato CE Brazil beatriz.castro022015@gmail.com; 5 Universidade Regional do Cariri (URCA), Crato-CE, Brasil. E-mail: edilma.rocha@yahoo.com.br Universidade Regional do Cariri Universidade Regional do Cariri Crato CE Brazil edilma.rocha@yahoo.com.br; 6 Universidade Regional do Cariri (URCA), Crato-CE, Brasil. E-mail: evanira.maia@urca.br Universidade Regional do Cariri Universidade Regional do Cariri Crato CE Brazil evanira.maia@urca.br; 7 Universidade Regional do Cariri (URCA), Juazeiro do Norte-CE, Brasil. E-mail: geycyenf.ga@gmail.com Universidade Regional do Cariri Universidade Regional do Cariri Juazeiro do Norte CE Brazil geycyenf.ga@gmail.com

**Keywords:** Violência contra a Mulher, Teoria de Enfermagem, Assistência Perinatal, Violence Against Women, Nursing Theory, Perinatal Care, Violencia contra la Mujer, Teoría de Enfermería, Atención Perinatal

## Abstract

**Introdução:**

A violência obstétrica pode ser do tipo física, verbal, psicológica, sexual e negligência da assistência. Não utilização de medicação analgésica, tratamento grosseiro, privação do direito de acompanhante durante o parto, procedimento sem o consentimento da parturiente são exemplos de violência, que está cada vez mais presente e de forma velada nos serviços de saúde brasileiro. Objetivou^- se^ analisar relatos de puérperas sobre violência obstétrica à luz da Teoria da Diversidade e Universalidade do Cuidado Cultural.

**Materiais e Método:**

Estudo transversal, abordagem qualitativa, desenvolvido em estratégias de Saúde da Família com 10 puérperas. A coleta de dados foi realizada através de entrevista semi-estruturada cujos resultados foram organizados e adaptados ao modelo *Sunrise.*

**Resultados:**

A maioria das participantes eram jovens, casadas/união estável, primíparas e com parto vaginal. Na adaptação do modelo, considerando seus conceitos, observou-se ausência de conhecimento do parto/trabalho de parto; medo; violência perpetrada contra as mulheres resultantes da ausência de comunicação, desumanização, exposição do corpo e desconforto, repercutindo em cuidado fragilizado, com insatisfação frente ao serviço de saúde.

**Discussão:**

A violência obstétrica é comum no cenário brasileiro, acontecendo muitas vezes de forma velada, pois as parturientes não conhecem sobre o assunto, bem como, seus direitos.

**Conclusão:**

A violência obstétrica aconteceu por meio do caráter sexual, físico, psicológico e institucional, tornando o ato de parir algo temoroso, resultante do medo, falhas na comunicação e cuidado fragilizado.

## Abstract (en)

**Introduction:**

Obstetric violence can involve physical, verbal, psychological and sexual abuse, and neglect of care. Refusal to administer pain relief, disrespectful treatment, deprivation of women’s right to be accompanied during childbirth, medical interventions without their prior consent are some examples of this violence, which is an increasingly present but hidden practice in healthcare services in Brazil. This study aims to analyze the narratives of labouring women on obstetric violence in the light of the theory of culture care diversity and universality.

**Materials and Methods:**

A qualitative cross-sectional study developed in the light of the Family Health Strategy was conducted with ten labouring women. Data was collected through a semi^- st^ructured interview with results organized and adapted to the Sunrise Model.

**Results:**

Most participants were young, married/in a stable relationship, primiparous with vaginal delivery. Based on the model principles, the lack of knowledge about childbirth/labor, medication, violence against women resulting from miscommunication, body exposure and discomfort leads to weakened care provision and dissatisfaction with health services.

**Discussion:**

Obstetric violence is a common practice in the Brazilian scenario that often occurs covertly due to the lack of knowledge about its existence and childbirth rights as labouring women.

**Conclusions:**

Obstetric violence involves sexual, physical, psychological and institutional abuse, making the act of giving birth seem frightening due to fear, lack of communication and weakened care provision.

## Resumen

**Introducción:**

La violencia obstétrica puede ser de tipo física, verbal, psicológica, sexual y de negligencia asistencial. La negativa a administrar medicamentos analgésicos, el trato brusco, la privación del derecho a estar acompañada durante el parto, el procedimiento sin el consentimiento de la parturienta son una muestra de esta violencia, cada vez más presente, pero de forma disimulada en los servicios asistenciales en Brasil. El objetivo es analizar los relatos de las parturientas sobre la violencia obstétrica a la luz de la teoría de la diversidad y universalidad de los cuidados culturales.

**Materiales y Métodos:**

Estudio transversal con enfoque cualitativo desarrollado a la luz de la política de Estrategia de Salud de la Familia con diez parturientas. Los datos se recolectaron a través de una entrevista semiestructurada y los resultados se organizaron y adaptaron al modelo de sol naciente.

**Resultados:**

La mayoría de las participantes eran jóvenes, casadas/en unión estable, primíparas y con parto vaginal. En la adaptación del modelo, a partir de sus conceptos, se observó la falta de conocimiento del parto/trabajo de parto, la medicación, la violencia perpetrada contra las mujeres resultante de la falta de comunicación, deshumanización, exposición del cuerpo y descontento, lo que repercute en un cuidado debilitado con insatisfacción frente al servicio de salud.

**Discusión:**

La violencia obstétrica es común en el escenario brasileño que a menudo ocurre de forma disimulada, debido al desconocimiento de su existencia y de sus derechos como parturientas.

**Conclusión:**

La violencia obstétrica pasa por el carácter sexual, físico, psicológico e institucional, lo que hace que el acto de dar a luz se vea como algo aterrador, producto del miedo, la falta de comunicación y la atención debilitada.

## Introdução

A violência é um problema que afeta a sociedade e por ser um fenômeno complexo, pode deixar sequelas irreparáveis^1^. A Organização Mundial de Saúde define violência como um ato de caráter intencional, com uso de força ou poder físico, ameaça ou real, contra si mesmo, outra pessoa ou contra um grupo, resultando ou não em ferimentos, morte, danos psicológicos, malformação ou privação^1^.

Os atos violentos ocorrem, sobretudo contra mulheres, nos lares, locais de trabalho e até mesmo em instituições sociais e de saúde^2^. Estima-se que a violência contra a mulher cause mais mortes na faixa etária de 15 a 44 anos do que câncer, acidentes de trânsito, guerras e malária^2^. Dentre as principais formas de violência estão assassinatos, estupros, abusos físicos, sexuais e emocionais, prostituição forçada, mutilação genital e violência racial^2^, sendo na maioria dos casos motivada por questões de gênero^3^.

Dentre aquelas que ocorrem nos serviços de saúde, a violência obstétrica é definida, principalmente, pela negligência na assistência, discriminação social, violência verbal (tratamento grosseiro, ameaças, gritos) e física (não utilização de medicação analgésica quando tecnicamente indicada)^3^^-^^5^.

Observa-se também a prática de violência psicológica, uso inadequado de tecnologias, adoção de procedimentos durante o ciclo gravídico-puerperal sem o consentimento explícito e informado da gestante/parturiente, proibição de um acompanhante, ferindo-se os princípios dos direitos individuais das mulheres^3^^-^^5^. O descumprimento de normativas importantes, também são percebidas em estudo com mulheres sobre violação dos direitos de parturientes^5^. Essa forma de violência é uma realidade em diversos países e em alguns estados brasileiros, na qual o uso inapropriado de autoridade por parte dos profissionais de saúde em relação ao corpo e à sexualidade das mulheres tem ocorrido frequentemente durante o parto e o pós-parto^6^.

Diante do exposto, a respeito da violência obstétrica, evidencia-se que a prestação de cuidados profissionais parece não ser sensível às necessidades deste grupo populacional, tratadas por vezes como recipientes de discriminação e práticas desumanizadas^7^, ainda culturalmente aceitas. Esses eventos conduzem a busca de novos caminhos para a prestação de cuidados assistenciais que vão de encontro às idiossincrasias do grupo em questão e que levem em consideração aspectos culturais envolvidos na prestação da assistência em saúde.

Assim, com vistas a refletir sobre a prestação de cuidados frente às particularidades dos indivíduos, famílias ou grupos, a Teoria da Diversidade e Universalidade do Cuidado Cultural (TDCUCC), proposta por Madeleine Leininger, aborda a necessidade de o profissional considerar o contexto cultural no qual o indivíduo se encontra inserido, propiciando cuidados harmônicos à aquela identidade local^8^. A TDUCC possui cinco conceitos que a sustentam, sendo eles a cultura, visão de mundo, contexto ambiental, cuidado e saúde^9^.

A cultura se refere ao conjunto de crenças, valores e normas que orientam o pensamento de determinado grupo social^9^. A visão de mundo volta-se para o modo como esses indivíduos entendem o mundo, sendo influenciada por aspectos econômicos, culturais, educacionais, dentre outros^9^.

O contexto ambiental se configura como uma experiência, acontecimento ou outra vivência particular, seja ela emocional e/ou física, no qual o indivíduo irá atribuir um sentido a experiência^9^.

Sobre o cuidado, este é permeado pelo apoio, auxílio ou suporte ofertado para que os sujeitos possam melhorar sua condição de vida ou ainda enfrentar devidas situações^9^. Por fim, a saúde é tida como o estado de bem-estar, culturalmente definido, permitindo os indivíduos e grupos sociais realizarem e valorizarem suas práticas de maneira satisfatória^9^.

O Modelo Sunrise possibilita a operacionalização da TDUCC e configura-se como uma representação sistemática visual dos principais componentes da teoria e suas inter-relações, permitindo um aprofundamento da compreensão desses componentes e possibilitando ainda visualizar o significado de cuidado para múltiplas culturas^10^.

Destacam-se que os fatores responsáveis por reações violentas podem ser modificados, sejam eles derivados de atitudes e comportamentos ou de condições políticas, econômicas, sociais e culturais^1^. Nesse sentido, acredita-se que por meio desse estudo, os profissionais de saúde possam refletir sobre suas práticas assistências a mulher em processo parturitivo, mudando seu comportamento direcionado as necessidades dessa mulher e orientando as gestantes desde o pré-natal quanto às atitudes que são consideradas violência obstétrica.

Assim, na perspectiva de contribuir com a discussão do tema, objetivou-se analisar os relatos de puérperas sobre violência obstétrica identificadas no cuidado obstétrico, à luz da Teoria da Diversidade e Universalidade do Cuidado Cultural.

A escolha da teoria em questão e do Modelo Sunrise se deu em decorrência destes fornecerem auxílio significativo no entendimento quanto à necessidade de prestação de um cuidado culturalmente congruente, sensível, que vai de encontro aos padrões de vida e as necessidades culturais de um grupo ou indivíduos, permitindo reflexões acerca da realidade das práticas clínicas em discussão.

## Materiales y Métodos

Estudo transversal, descritivo, com abordagem qualitativa, desenvolvido em três equipes de Saúde da Família (eSF), localizadas na zona urbana de um município da região metropolitana do Cariri, Ceará, Brasil. Essas equipes foram escolhidas devido ao bairro em que estavam inseridas possuir maior quantitativo populacional do município.

O estudo foi apresentado ao gestor municipal de saúde, concedendo-se anuência para realização nas unidades mencionadas acima.

A coleta de dados foi realizada entre os meses de abril e maio de 2017. A identificação das mulheres que estavam no puerpério foi realizada por meio de contato pessoal com as enfermeiras das equipes, que viabilizaram o acesso aos prontuários para confirmação dos dados. Posteriormente, fez-se contato pessoal com os respectivos Agentes Comunitários de Saúde (ACS’s) das eSF’s, que ajudaram a agendar previamente visitas domiciliares com as mulheres selecionadas, mediante a disponibilidade de cada uma.

Foram visitadas 15 puérperas adscritas nas eSF´s. Um total de 10 corresponderam aos critérios de inclusão: estar no puerpério; idade igual ou superior a 18 anos; parto ocorrido em uma maternidade e acreditar ter sofrido violência obstétrica. Foram excluídas do estudo cinco mulheres que sofreram intercorrência com risco de vida durante o trabalho de parto e adoção de recursos traumáticos (a exemplo da utilização de fórceps), que poderiam ser associados pelas puérperas como violência obstétrica. No domicílio da puérpera, a pesquisadora explico sobre o objetivo da pesquisa, e em acordo, a participante assinou o Termo de Consentimento Livre e Esclarecido, sendo a entrevista realizada no local. As entrevistas foram encerradas após se identificar o ponto de saturação teórica.

Para a coleta de dados adotou-se a técnica de entrevista semi-estruturada, composto por três partes: a primeira com questões fechadas referentes aos dados sociodemográficos e a segunda e terceira com questões abertas relacionadas ao histórico da última gestação e aos aspectos do parto e da violência obstétrica, respectivamente.

As entrevistas foram gravadas em mídia digital e, posteriormente, transcritas na íntegra com duração média de 40 minutos cada. Para garantir o anonimato das participantes, estas foram identificadas com a letra P por designar puérperas, seguida de número ordinal de 1 a 10 (ex. *P1, P2, ...P10).*

Na organização e apresentação dos dados, optou-se por realizá-las de acordo com o modelo Sunrise, proposto por Leininger^10^. Seguiram-se as seguintes etapas: 1^- Se^leção dos registros (falas) de acordo com os conceitos cultura, visão de mundo, contexto ambiental, cuidado e saúde; 2^- Ag^rupamento dos dados mais significativos e relevantes por conceito e 3-Construção da adaptação do Modelo Sunrise com base em ideias centrais dos agrupamentos.

Quanto à análise dos dados advindos da adaptação do Modelo *Sunrise*, a mesma ocorreu à luz do referencial da Teoria da Diversidade e Universalidade do Cuidado Cultural, associado à literatura científica pertinente à temática. A pesquisa foi submetida e aprovada pelo Comitê de Ética em Pesquisa da Universidade Regional do Cariri (CEP/URCA) com parecer aprovado nº 1.92.672.

As informações validadas, acerca da resposta das participantes, foram exportadas para o pacote estatístico Stata/MP versão 14.0. para processamento de dados. O banco de dados foi armazenado no Mendeley Data^11^.

## Resultados

### Caracterização dos participantes do estudo

Participaram do estudo 10 puérperas. Identificou-se que a maioria (seis) das puérperas eram jovens, com idade entre 18 a 23 anos. Seis estavam casada/união estável, duas solteiras e um separada do parceiro, três moravam com sua família/genitores. Em relação ao número de filhos, seis eram primíparas e oito realizaram tipo de parto vaginal. Em relação à escolaridade, duas participantes conseguiram concluir o ensino médio, cinco tinham ensino médio incompleto, uma possuía ensino fundamental incompleto e duas tinham ensino superior incompleto.

Quanto à ocupação das participantes, prevaleceu do lar, empregadas domésticas, estudantes e uma autônoma. Das que possuem alguma atividade remunerada, nenhuma a exerce com vínculo formal. A renda familiar variou entre 900 a 1.800 reais, sendo que o maior provedor é o companheiro.

No Quadro 01 apresenta-se o agrupamento dos registros mais significativos por conceito a partir da adaptação do Modelo Sunrise de Leninger.

**Quadro 01:** Significado atribuído pelas puérperas, a partir de suas vivências, ao cuidado obstétrico recebido adaptado ao modelo Sunrise, Ceará, Brasil, 2017.

Conceito	Fala da puérpera	Tipo de violência
Cultura	[...] eles diziam que ia ser um parto induzido, eu achava que não ia ter passagem suficiente, estava com medo de ir pra cesárea. (P2)	Física (administração de medicamentos para acelerar as contrações uterinas sem o consentimento materno) e Psicológica (adoção de procedimentos sem o consenti^- me^nto explícito e informado da parturien^- te^; ausência de informação e suporte emocional)
	Eu não sabia não, que eu podia conhecer o hospital antes de ganhar não. Pensei que só podia ir quando fosse pra ganhar. (P10)	Psicológica (ausência de informação)
	Eu tive muito medo na hora, medo da minha filha morrer. Acho que é comum a gente sentir medo nessa hora. (P4)	Psicológica (ausência de informação e suporte emocional)
	Acho que toda mulher tem medo quando vai ter um filho, seja ele normal ou cesáreo. Passa um monte de coisa na sua cabeça. (P9)	Psicológica (ausência de suporte emocional)
Visão de Mundo	Eu acho que acontece sim violência nos serviços de saúde [...] e com as grávidas também. Principal^- me^nte quando os médicos não trata bem os pacientes, realizam os procedimentos sem avisar [... (P3)	Psicológica (adoção de procedimentos sem o consentimento explícito e informado da parturiente e ausência de informação) e Verbal (tratamento grosseiro)
	[...] você ta num lugar, e as pessoas costumam, as vezes, se engrandecer daquela profissão, querem ser superior a você e agente ta depen^- de^nte dela. Então quando não trata bem, o medo aparece, medo de ser maltratada. (P7)	Psicológica (humilhação); Discriminação social e Verbal (tratamento grosseiro)
	Percebi que os profissionais eles não dão muita atenção, não são de conversar não. Eles só dizem o que você tem que fazer e pronto, te deixa lá (P8)	Psicológica (uso inadequado de tecnologias, adoção de procedimentos sem o consenti^- me^nto explícito e informado da parturien^- te^; ausência de informação e parturiente passiva no parto)
	[...] muitas vezes eles (os profissionais) não repassam as informações, e quem ta lá internada quer saber o que ta acontecendo, se ta tudo bem. Tem deles que respondem com ignorância, mas é trabalho deles responder. (P3)	Psicológica (ausência de informação) e Verbal (tratamento grosseiro)
Contexto Ambiental	Me senti mal durante o parto, porque eram muitos estagiários olhando pra mim, você ali numa posição nada confortável, com dor e um monte de gente olhando pra você [...] (P2)	Violação da privacidade; Física (não utilização de medicação analgésica quando tecnica^- me^nte indicada) e Psicológica (uso inade^- qu^ado de tecnologias e parturiente passiva no parto)
	Eu tive a minha filha a noite, eu gritava muito de dor, e eu ficava sozinha, pra não dizer que não tinha ninguém tinha outra mulher pra ganhar também [...] Ter esse menino na noite foi horrível, a pessoa fica só lá naquela sala. (P6)	Física (não utilização de medicação analgésica quando tecnicamente indicada) e Psicoló^- gi^ca (privação do direito de acompanhante e abandono)
	Não eu não tive acompanhante, minha sobrinha queria assistir, mas o pessoal do hospital disse que ela não podia, que só quem podia era o pai, só que meu marido não tinha coragem de ir, então fui sozinha mesmo. (P7)	Psicológica (privação do direito de acompan^- ha^nte de livre escolha da parturiente)
	É horrível, porque eu fiquei exposta [...] Eu chorava bastante, acho que chorava de dor e chorava de vergonha. (P4)	Violação da privacidade e Física (não utilização de medicação analgésica quando tecnica^- me^nte indicada)
Cuidado	Quando eu ganhei o menino, já deu infecção em mim no outo dia [...] me mandaram pra casa sem eu tá melhor [...] Ai voltei pro hospital novamen^- te^. A médica que tava lá fez um toque em mim e viu que tinha um ponto inflamado [...] Ai resolve^- ra^m me internar, fiquei três dias lá, tomando antibiótico. (P1)	Negligência na assistência e Recusa de internação (no primeiro momento)
	Não, eu não escolhi o tipo de parto não [...] Eu queria muito ter normal, mas não deu [...] (P8)	Psicológica (parturiente passiva no parto e ausência de informação)
	[...] as contrações também era muito forte, cada vez mais forte. [...] não, eles não mandavam eu fazer nada pra aliviar a dor.(P4)	Negligência na assistência; Física (não utilização de medicação analgésica quando tecnicamente indicada e Psicológi^- ca^ (parturiente passiva no parto e ausência de informação)
	E eu acho que o médico devia prestar mais atenção na gente, porque eles manda a gente ficar deita^- da^ e esquece a gente lá. (P3)	Negligência na assistência e Psicológica (parturiente passiva no parto e ausência de informação)
	[...] não eu não escolhi a posição em que meu filho ia nascer?! Podia?! [...] eu tive ele deitada mesmo. (P5)	Psicológica (parturiente passiva no parto e ausência de informação)
	Eu não vi (a episiotomia). Eu não tive coragem de ver. Quer dizer eu não perguntei e nem me disse^- ra^m, eu vi nos papeis que eles entregam. É uma coisa que era pra eles dizerem. (P2)	Psicológica (ausência de informação e adoção de procedimentos sem o consentimento explícito e informado da parturiente)
Saúde	Eu não fiz o acompanhamento completo do pré-na^- ta^l porque não tinha médico nem enfermeiro[...] mas graças a Deus não teve nenhum problema. (P10)	Negligência na assistência
	Eu passei três dias sem amamentar, que foi o tempo que fiquei internada. O hospital não quis deixar o meu filho comigo. [...] as vezes eu desmamava um pouco de leite e minha irmã ia levar pra ele mamar. [...] graças a Deus ele não deixou de mamar. [...] mas a minha maior alegria foi chegar em casa bem e ver ele bem também, graças a Deus. (P1)	Psicológica (afastamento da mãe e do filho)
	Eu queria que minha filha nascesse logo. E graças a Deus eu não senti nada durante a cirurgia. (P8)	Psicológica (ausência de suporte emocional)

Destaca-se que cada categoria foi construída com base no conceito proposto pelo modelo *Sunrise*, alocando-se as falas relacionadas ao conceito, bem como, considerando as formas de violência sofridas por essas mulheres. Assim, na categoria referente a “cultura”, registrou-se o conhecimento que as puérperas tinham em relação ao parto e aos atos de violência nesse momento, destacando-se que as vezes existe uma dificuldade por parte dessas mulheres se reconhecerem vítimas de violência, devido a naturalização dessas práticas.

A categoria “visão de mundo” considerou a percepção sobre os tipos de violência obstétrica praticados contra elas durante o parir, destacando-se um atendimento pautado na ausência de comunicação e desumanização, revelando-se a existência de profissionais desumanizados, sendo essa a concepção obtida do SUS.

A categoria “contexto ambiental” apresentou relatos de parturientes sozinhas e sem direito a acompanhante, sendo esta situação uma forma de violência e violação de direitos, além da situação desconfortável e de exposição do corpo a qual estavam submetidas. Ainda, todos os comentários reforçam experiências e sentimentos negativos em meio ao ambiente hospitalar e isso pode gerar traumas futuros.

Na categoria “cuidado”, a ideia central foi a percepção de uma assistência desatenta as necessidades da parturiente, refletindo um cuidado fragilizado, obrigando a mulher a adotar postura passiva ao parir. Por fim, a categoria“saúde” expressou a opinião dessas mulheres acerca do serviço de saúde prestado a elas durante toda a gestação e o seu desfecho, observando-se a presença de insatisfação da assistência recebida.

**Figura 1:**
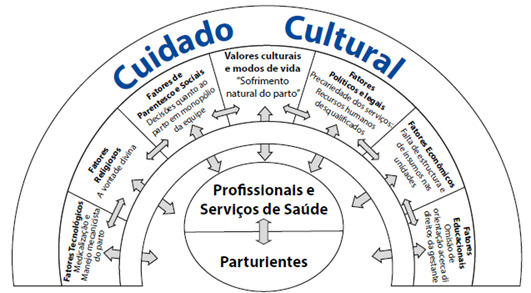
Adaptação do Modelo Sunrise. Associação entre as convicções políticas, educacionais, econômicas, religiosas e culturais e valores que influenciam os cuidados de saúde a parturientes, Ceará, Brasil, 2017.

Na Figura 1 têm-se o resultado da construção da adaptação do Modelo *Sunrise*, de modo que dos dados mais significativos e relevantes por conceito foram extraídas as ideias mais recorrentes, traduzidas em expressões chaves.

Fonte: Adaptação de Sunrise Model ^13^

## Discussões

O parto é um momento de experiência singular, no qual a mulher vivencia a consolidação de uma expectativa alimentada desde a descoberta da concepção. Nesse sentido, o parto é um evento que integra o rol das experiências humanas mais significativas para os envolvidos. No entanto, muitas vezes, é permeado pela violência, perpetrada justamente por quem deveria ser o principal ator no cuidado, o profissional de saúde^12^ e que tem como fator associado à sua ocorrência, aspectos culturais. Assim, a bagagem cultural que a mulher traz consigo sobre o ato de parir e os aspectos culturais envolvidos nas condutas profissionais irá influenciar toda assistência, de modo que essas influências e suas repercussões podem se dar de maneiras distintas.

Leininger aponta a cultura como sendo um componente essencial a ser considerado na prestação dos cuidados, por a mesma está permeada de comportamentos e práticas relativas ao estilo de vida, guiando o indivíduo ou determinado grupo em suas ações e pensamentos. Na teoria, identifica-se que cada indivíduo constrói um modo como deseja ser cuidado^9^.

Assim, quando identificado medo como uma das ideias centrais nos relatos referentes à vivência do parto, aponta-se uma cultura social em que o ato de parir aproxima-se da morte, seja ela da mulher ou do recém-nascido. Parir ganha um significado que se entrelaça ao receio de se não ter condições fisiológicas que permitam um parto normal, sendo necessário recorrer a um procedimento cirúrgico.

Ressalta-se que embora o Brasil venha adotando medidas propostas pela Rede Cegonha para minimizar a mortalidade materna, observa-se de acordo com os dados do Sistema de Informação sobre Mortalidade (SIM) que em 2015 houveram 1.738 notificações de morte materna envolvendo óbitos por problemas relacionados à gravidez, ao parto ou ocorridos até 42 dias depois deste e que, embora em 2016 tenham sido registrados 1.463, o que representa uma queda de 16% em relação ao ano anterior, este ainda é um número alto^13^.

Sabe-se que até o século XVII, o parto era um momento extremamente feminino, no qual a parturiente, a parteira e as parentes mais próximas ajudavam na condução desse momento, na residência da mulher, sem preocupações com condições fisiológicas. Com a modernização, bem como, o avanço da medicina, o ato de parir saiu do cenário domiciliar e foi para o hospitalar, com o intuito de assistir melhor a parturiente e o seu filho, no entanto, tornou-se um processo medicalizado, no qual a parturiente passou de protagonista a objeto^14^.

Essa falta de conhecimento também pode estar associada à baixa escolaridade, dado que foi identificado neste estudo, associado à baixo status econômico, e que pode influenciar na construção desse conjunto de valores, práticas e conhecimento apreendido, partilhado e transmitido dentro do mesmo grupo, configurando-se o conceito de cultura. Além da cultura de morte, existe um medo associado à precarização dos serviços de saúde.

Ainda, considerando-se o perfil das puérperas do estudo, destaca-se que na realidade das maternidades brasileiras, a violência obstétrica é determinada por questões de gênero, ou seja, ser mulher pobre e de baixa escolaridade a coloca em posição de desigualdade na relação hierárquica frente aos profissionais de saúde, em que a mulher é tratada como objeto de intervenção profissional e não como protagonista de suas próprias decisões e atos^3^^,^^6^^,^^15^. Nesse caso, prevalece as intervenções e procedimentos técnicos e tecnológicos, cujo profissional da saúde executa seu serviço, finaliza a assistência e não interage com a paciente enquanto sujeito^3^.

Com base nos pressupostos da Teoria da Diversidade e Universalidade do Cuidado Cultural, cabe a equipe a identificação desses aspectos culturais no seu processo de assistência, não no intuito de uma intervenção que remodela, mas sim na busca de uma reflexão sobre como o cuidado prestado atinge as crenças e valores já presentes no modo de vida da parturiente, reforçando-os ou refutando-os, sobre como os aspectos educacionais, econômicos e sociais podem estar sustentando essas concepções culturais, e ainda, sobre como a imposição da cultura pessoal sobre essa mulher se configura como uma violência, compreendendo que o indivíduo encontra-se inserido em um contexto que alimenta e sustenta suas crenças.

A visão de mundo constitui-se como um quadro ou atitude de valor que emerge como fruto de uma observação do mundo e das vidas dos indivíduos ou grupos sociais^9^. No momento do parto, a mulher observa todo o cenário que a cerca, a maternidade é submetida a sua ótica, bem como os profissionais que estão presentes nesse espaço e suas condutas. Esses elementos se consolidam em uma visão permeada pelos significados atribuídos ao seu entendimento.

A falta de vínculo entre profissional e parturiente, implica em uma visão de mundo negativa frente à maternidade. Assim destaca-se a ausência de comunicação, contida na falta de diálogo constante, deixando por vezes a gestante/puérpera sem informações concernentes ao ato de parir e quadro clínico, o que gera uma ideia de abandono durante a internação. Considera-se ainda, a necessidade de se informar os procedimentos a serem adotados previamente, a partir do uso de uma linguagem pautada no respeito, que não venha a ferir verbalmente essa mulher.

A comunicação efetiva pela equipe é importante, especialmente por profissionais que atuam diretamente na assistência a estas mulheres, como os profissionais médicos e equipe de enfermagem. Dentre os profissionais de saúde, o médico ocupa o topo dessa relação hierárquica, sendo sua autoridade não fundamentada apenas por possuir conhecimentos científicos e tecnológicos, mas também, pela visão de mundo associada à valores que o caracterizam como o profissional referência na assistência. Assim, a sociedade se coloca em uma situação de dependência frente aos cuidados em saúde e, neste meio, o poder médico é produzido pela comunicação entre os sujeitos sociais em condições de desigualdade^16^. Essa visão de mundo pode justificar a percepção das participantes do estudo frente ao abandono sentido diante da ausência e da falta de comunicação destes profissionais, o que caracteriza desumanização da assistência.

De fato, quanto à desumanização, ela se desenvolve através de algumas nuances, podendo^- se^ destacar os relatos que afirmam distanciamento entre a equipe e a mulher, sendo os profissionais desatenciosos, sem diálogo e com omissão de informações. Há ainda as práticas mecanicistas, desprovidas de humanização, de modo que o tratamento recebido configura-se como maus tratos ou como receio de serem vítima desses, gerando-se uma atmosfera de medo. Tais condições revelam muitas vezes uma violência velada, silenciosa.

Conforme a Teoria da Diversidade e Universalidade do Cuidado Cultural constata-se que a visão de mundo se relaciona a autoestima e a identidade cultural dos indivíduos ou grupos sociais específicos, nos quais há um desnudamento de suas perspectivas de vida^17^, ou seja, a pessoa se priva daquilo que traz consigo quando adentra aquele novo mundo, nesse caso, o ambiente hospitalar.

Nesse sentido faz-se necessário prestar uma assistência que corrobore com a estruturação de um serviço que seja adequado a paciente e ao contexto em questão, os profissionais devem agir de modo que gerem na gestante e/ou parturiente uma visão de mundo que aponte qualidade na assistência.

Uma das formas de violência obstétrica, a psicológica, é compreendida como toda ação verbal ou comportamental que cause na mulher sentimentos de inferioridade, vulnerabilidade, abandono, instabilidade emocional, medo, desconforto, acuação, insegurança, dissuasão, ludibriamento, alienação, perda de integridade, dignidade e prestígio. Podem ser citados como exemplos ameaças, mentiras, chacotas, piadas, humilhações, grosserias, chantagens, ofensas, omissão de informações, informações prestadas em linguagem pouco acessível, desrespeito ou desconsideração de seus padrões culturais^5^^,^^6^.

O contexto ambiental que se faz presente no momento do parto pode ser decisivo sobre quais impressões e significados aquela mulher irá atribuir a sua experiência. Destaca-se que aqui se toma como referência para contexto ambiental a totalidade de um acontecimento, conferindo sentido as expressões humanas associadas às interações e dimensões envolvidas^9^.

Os relatos discorrem sobre mulheres que desconhecem os mecanismos do parto e os direitos das gestantes. Elas chegam as unidades hospitalares desprovidas de informações valiosas que as instrumentariam a adotar uma postura ativa diante das condutas da equipe de saúde.

Voltando-se para o ambiente hospitalar, cenário esse de ocorrência do parto das participantes do estudo, a associação das ideias centrais acaba por revelar um espaço que se configura como hostil e propício à identificação da violência obstétrica, no qual, no ato de parir, dentre outros, a mulher sentiu-se desconfortável e exposta em estar sob o olhar de acadêmicos de saúde, o que gerou uma sensação de vergonha e mal estar associada ao quadro de dor que já se fazia presente.

Assim, em hospitais escola, é rotineiro existir vários discentes juntos ou em sequência para realizar procedimentos. Não sendo bastante a exposição e o incômodo na realização desses procedimentos, a parturiente não é informada dos nomes, da qualificação, da necessidade e riscos do procedimento, ou mesmo das informações sobre a progressão do seu próprio trabalho de parto. Além disso, em nenhum momento é solicitado o seu consentimento para permitir ou não os procedimentos e a presença de estudantes^5^.

Em relação a esta realidade, em Sobral, localizado no Ceará, foi aprovada em 15 de março de 2016, a Lei Nº 1550, que proíbe a prática de violência obstétrica, exemplificando algumas situações que podem caracterizar essa violência, dentre elas submeter a mulher a procedimentos dolorosos, desnecessários ou humilhantes, ferindo sua privacidade; executar qualquer procedimento sem o consentimento prévio e explicação sobre a necessidade deste ser realizado e submeter a mulher e/ou o bebê a procedimentos para treinar discentes^18^.

Destaca-se que a recusa de um acompanhante durante o pré-parto, o parto e o pós-parto configura-se também como uma violência^9^. Mesmo sendo um direito garantido pela Lei 11.1008/2005 a essa mulher, muitas vezes nem ela, nem seus parceiros a conhecem^19^. No entanto, embora o usuário desconheça tal lei, o serviço de saúde precisa cumpri-lá, garantindo que os direitos de saúde com qualidade não sejam vetados. A presença do acompanhante pode ajudar em ações que resultem no conforto físico, como por exemplo, auxílio na deambulação, no banho e nos exercícios de respiração, dentre outros e, no conforto emocional a parturiente^12^.

Cabe ao profissional de saúde desde o momento da realização das consultas de pré-natal, orientar a mulher sobre os seus direitos, dentre eles, o direito de ter um acompanhante de sua escolha durante todo o processo de parto, além do direito que esta possui de conhecer a maternidade em que ocorrerá o seu parto^19^. No momento da internação da mulher, a equipe de saúde da maternidade deve estar preparada para receber, estimular e orientar o acompanhante, promovendo sua participação em todas as dimensões do apoio parturiente^12^.

Nos moldes da Teoria da Diversidade e Universalidade do Cuidado Cultural, o ambiente não se limita as dimensões físicas as quais comumente se conhece. A experiência vivenciada é tida em sua totalidade, com aspectos relacionados às interações sociais, as interpretações e expressões humanas envolvidas. A soma destes e outros pontos levam a totalidade da percepção do contexto ambiental^9^.

Considerando esse conceito de contexto ambiental que se faz presente na produção de Leininger, fica expresso a necessidade da equipe se comunicar, preservar a imagem da parturiente, solicitar a sua permissão e considerar as suas necessidades e queixas, sem minimizá-las ou ainda subestimá-las, visto que as puérperas alegaram uma desacreditação por parte da equipe no que elas expressavam durante o parto. É preciso que os profissionais reconheçam o poder que suas práticas possuem de alterar uma vivência, bem como a influência do ambiente no qual prestam sua assistência, tendo como base a noção de que esses aspectos irão influenciar na totalidade daquele evento.

Observa-se atualmente uma atenção maior por parte do Ministério da Saúde em promover a qualidade na atenção ao parto, no entanto, ainda se faz necessário uma mudança nas práticas assistenciais observando os protocolos instituídos pelos serviços de saúde. Essa transformação deve conferir a parturiente a condição de ativa e participante do processo parturitivo, visto que ainda acontecem atitudes consideradas violentas, com regras e rotinas que valorizam a assistência profissional e não o valor da experiência da parturiente^12^^,^^20^.

Na análise dos relatos associado ao termo cuidado, depara-se com práticas prescritivas, além disso, identificou-se a ausência dos profissionais em alguns momentos tidos como importantes e a adoção de uma conduta terapêutica que não vai de encontro à necessidade da parturiente, não considerando as suas queixas.

Percebe-se que, além das formas de violência obstétrica sofridas por essas puérperas já citadas, a violência também pode ser física, que é entendida como as ações que incidem sobre o corpo da mulher, que interfiram ou causem dor ou dano físico e sem recomendação baseada em evidências científicas, tendo-se como exemplos a privação de alimentos, interdição à movimentação da mulher, tricotomia (raspagem de pelos), manobra de Kristeller, uso rotineiro de ocitocina, cesariana eletiva sem indicação clínica e não utilização de analgesia quando tecnicamente indicada^4^^-^^6^.

Outra forma de violência obstétrica é a ausência de informação durante o parto, bem como tornar a parturiente passiva no ato de parir. Assim, o cuidado prestado pode não valorizar o conhecimento sobre o próprio corpo e essa condição talvez ocorra pela expectativa das entrevistadas, de que o profissional saiba tudo o que deve ser feito e, portanto, a ele caiba a responsabilidade pelas decisões tomadas^5^.

As puérperas desse estudo alegam não terem feito parte do processo de tomada de decisão quanto às condutas adotadas na assistência ao parto, como por exemplo, o tipo de parto e além disso, os relatos ainda realçaem que a mulher está constantemente recebendo intervenções sem um conhecimento prévio, como por exemplo a episiotomia, e por vezes sem realizar questionamentos a respeito.

Sobre o caráter sexual da violência, podem ser citados a episiotomia, exames de toques invasivos, cesariana sem consentimento, ruptura ou descolamento de membranas sem consentimento informado, dentre outros^12^^,^^20^.

Os pressupostos da Teoria da Diversidade e Universalidade do Cuidado Cultural apoiados no Modelo *Sunrise* guiam os profissionais à prestação de um cuidado culturalmente congruente, na busca de um real estímulo e manutenção da saúde daquele cliente. A equipe deve identificar a necessidade do planejamento de cuidados, de modo a aproximar-se das reais necessidades do paciente, identificando se estes requerem da preservação, do ajustamento ou da repadronização/ reestruturação do cuidado cultural^9^.

O conceito de saúde vem sendo discutido ao longo das últimas décadas através de diversas óticas, assim, acentua-se que o conceito de saúde, nos moldes da TDUCC, volta-se para o estado de bem-estar que permite que o indivíduo ou grupos pratiquem suas atividades cotidianas de maneira satisfatória^9^.

As ideias identificadas nos relatos associados a esse conceito remetem a uma insatisfação, ou seja, negativação frente aos profissionais e serviços de saúde. Isto se deu devido à ausência da prestação de assistência a gestante ainda no pré-natal, por conta da falta de médico e enfermeiro que realizasse as consultas na unidade básica de saúde, além disso, a um distanciamento forçado da puérpera com o recém-nascido, o que gerou um sentimento de preocupação. Todas essas situações refletem em práticas de violência ao seu direito amplo de saúde.

Sobre o caráter institucional dessa violência, são entendidos como as ações ou formas de organização que dificultem, retardem ou impeçam o acesso da mulher aos seus direitos constituídos, sejam estes ações ou serviços, de natureza pública ou privada^5^. Pode-se destacar o impedimento do acesso aos serviços de atendimento à saúde, impedimento à amamentação, omissão ou violação dos direitos da mulher durante seu período de gestação, parto e puerpério, protocolos institucionais que impeçam ou contrariem as normas vigentes, dentre outros^21^.

Quanto à fé como resolutiva e elemento necessário para se ter saúde, ela também se apresentou como uma ideia recorrente nos relatos, atribuindo a Deus a melhora do quadro, a obtenção da alta e até mesmo a não ocorrência de complicações durante o parto. Essa mesma fé se consolidou como um instrumento de apoio durante o processo de internação. Desse modo, constata-se que esse estado de bem-estar é atribuído a uma concepção religiosa, ao componente da fé, não sendo citada a equipe como componente relevante nesse estado de bem-estar.

A Teoria da Diversidade e Universalidade do Cuidado Cultural traz recomendações para que os profissionais estejam aptos a assistir, apoiar e/ou facilitar o cuidado, bem como capacitar o cliente a participar do processo de cuidado, considerando suas experiências prévias. A prestação de um cuidado culturalmente congruente dá suporte para que o indivíduo, grupo ou família busque mecanismos para superar sua condição de vida, almejar melhorias e inclusive saber lidar com a morte e deficiências.

De maneira geral, a assistência prestada não se mostrou nos relatos das participantes do estudo como fator enriquecedor do estado de bem-estar, o que pode estar intimamente ligado às discussões anteriores, visto que houve a constatação de práticas revestidas por atos de violência obstétrica, resultando em um julgamento que não inclui o manejo da equipe como algo que incentiva a prática satisfatória de suas atividades, e consequentemente a sua saúde.

## Considerações Finais

ATeoriada Diversidadee Universalidadedo Cuidado Culturaldeusuporteteóricoemetodológico, guiando as discussões dos achados. Nesse sentido, observou-se que frente à cultura, mulheres podem não se reconhecerem enquanto vítimas de violência devido a sua naturalização. A visão de mundo da puérpera pode apontar para uma necessidade de atenção hospitalar, embora com carência no reconhecimento de seus direitos na maternidade. Já o contexto ambiental, no caso da puérpera vitimizada, pode revelar impactos negativos emocionais na assistência. O cuidado, mediante violência obstétrica, mostra-se fragilizado. Por fim, a saúde quando associado à vitimização obstétrica, implica em descrédito no serviço, com sua fragilização.

A partir dos resultados, o estudo pode guiar o profissional de saúde que presta assistência, desde o pré-natal e principalmente no parto, a uma reflexão sobre suas práticas de cuidado, o qual deve considerar o contexto em que essa parturiente está inserida, bem como seus valores culturais e crenças.

Como limitação, tem-se o fato de que a violência obstétrica é um assunto complexo, perpassado por várias dimensões, muitas vezes, por atos velados durante a assistência recebida no parto, além da falta de informação acerca desse assunto, sendo possível que as puérperas estudadas não consigam perceber todas as nuances, por envolver questões subjetivas.
